# Thyroidectomy in Inoperable Cancer

**Published:** 1909-10-02

**Authors:** 


### SPECIAL ARTICLE.

### THYROIDECTOMY IN INOPERABLE CANCER.

### WORK AT THE CENTRAL LONDON EAR AND THROAT HOSPITAL.

At Mr. Stuart-Low's clinic at the Central London Throat and Ear Hospital may be seen some very interesting cases of advanced cancer of the tongue, larynx, and adjacent parts, in which he appears to have arrested the disease at least for the present, and effected a considerable amelioration in the patient's condition by performing the operation of thyroidectomy. The hypothesis which led Mr. Stuart-Low tentatively to adopt this method of treatment supposes that excessive activity of the thyroid gland is a causal factor in the production of cancer. He suggests that such excessive activity of the thyroid induces a diminution of mucin in the tissues, a state of hypomyxia which he calls a precancerous condition. He points out that myxœdema and cancer do not occur together in the same individual, but that, on the other hand, enlarged thyroid, with over-activity of the gland, does occur in wasting diseases such as tuberculosis and cancer, and he suggests that the rapid pulse of cancer patients may be attributable to this over-activity. He thinks, too, that the rapid healing usually experienced in cancer cases indicates an active metabolism due to this increased activity of the thyroid.

### The First Case.

Such are some of the theoretical considerations which led him to try thyroidectomy in advanced inoperable cases of cancer. The first case was a man aged 63 with an epitheliomatous ulcer on the left side of the base of the tongue, spreading to the palatoglossal folds and adjacent floor of the mouth, and a large mass of glands on the left side of the neck. There was much induration and pain. On December 2, 1907, the whole of the left half of the tongue was removed down to the hyoid bone and the adjacent affected parts, and the thermo-cautery freely applied. The mass of glands was also removed. The patient did well for six weeks, but recurrence then took place. Sodium cinnamate injections proved quite ineffective. The left side of the floor of the mouth was full of ulcerated growth extending to the right base of the tongue, and at the seat of operation in the neck there was a mass of hard glands breaking down and discharging pus and blood. On February 14, 1908, the thyroid gland was excised and low tracheotomy performed under chloroform. The gland was found to be very vascular and adherent all round, and pathological examination showed a pronounced condition of hypertrophy. The patient made a good recovery, and walked out of the hospital at the end of a week. In twelve days the mass of glands in the neck was smaller, the discharge had almost ceased, there was less induration and no pain. The patient was able to swallow and talk much better, and the pulse was slower, being only 68. He was given three ounces of the elixoid of mucin daily. He gained a pound in weight each week for two months after the operation. In June, however, he developed septic pneumonia from the discharging ulcer in the mouth, and died on the 22nd. During the four months that he lived after the operation he developed no sign of myxœdema.

### Some Later Cases.

The second case was a man aged 45, who was shown at a meeting of the Laryngological Section of the Royal Society of Medicine on May 7, 1909, and was pronounced to be an inoperable case of epithelioma of the larynx. There was a large grey mushroom-like mass covering over and projecting into the larynx. Removal of about one-third of the growth gave considerable relief. The pathological report was a rapidly-growing epithelioma. On May 9 removal of the thyroid was attempted under cocaine anæsthesia, but obstruction from the growth necessitated tracheotomy, and the vessels to the gland were simply ligatured on both sides; the left half of the thyroid sloughed away in the dressings. The patient left the hospital in a fortnight. This patient now attends the hospital as an out-patient, and can be seen at the Clinic. He is much improved, he has gained 10 lbs. in weight, the growth has diminished, and he has no pain. He expresses himself as feeling much better since the operation.

The third case is a man, a painter, aged 58, with an epitheliomatous ulcer on the right side of tongue, with induration extending to the palate and the right side of pharynx. There was a large mass of glands in the neck. Pathological examination confirmed the diagnosis. On June 3, 1909, the left half of the thyroid was removed. It was found to be very vascular and adherent. Patient quickly recovered from the operation. He has been able to return to work. He has been quite free from pain, whereas before the operation the pain necessitated a hypnotic every night. The ulcer has healed, and the induration has diminished. He has gained half a stone in weight. The most interesting point in the case is the condition of the mass of glands in the neck. This became softer, and threatened to break down. It was therefore freely opened and a glairy mucoid fluid was discharged, which, on pathological examination was pronounced to consist chiefly of mucin. The inference is, therefore, that the removal of the thyroid has induced a myxomatous degeneration in these cancerous glands.

The fourth case was a man, aged 70, with a large excavated ulcer on the outer wall of the left side of the pharynx, with a hard mass of glands under the sternomastoid. He was losing weight, and complained of great pain. On June 16, 1909, the thyroid vessels were ligatured on both sides under local anæsthesia. The patient recovered well. He was free from pain. The glands became smaller, and he was able to swallow better; but he was in very poor and dirty condition, and he died a few weeks later.

The fifth case was a man, aged 65, with epithelioma of the soft palate, with a mass of glands in the neck. In July 1909 a partial thyroidectomy was performed. Since then the patient has improved in general condition; he is free from pain and the glands are smaller.

### Conclusions.

Thus altogether five cases have been treated by thyroidectomy, and of these all have for a time shown signs of improvement, and three of the cases are still living and under observation, and can be seen at Mr. Stuart-Low's Clinic. Their subsequent history will, of course, be of considerable interest. One of the most striking results in the whole story is the discharge of mucin from the mass of glands in the neck in the third case. This would certainly appear to lend support to the hypothesis of Mr. Stuart-Low that there is some connection and interaction between activity of the thyroid gland and cancerous growth. How great or how small that connection may be, and many other things, can only be revealed by further work and observation. Mr. Stuart-Low does not make any pretensions to a cancer-cure, he has only attempted a means of alleviating the suffering, and possibly prolonging the life of a few inoperable cases. How far removal of the thyroid may prove to be a remedial means of combating the disease only further experience can show. At any rate the theory raised by the work that has so far been done deserves recognition and attention at the hands of those who are interested in cancer research.

